# Algorithmic Assessment of Vaccine-Induced Selective Pressure and Its Implications on Future Vaccine Candidates

**DOI:** 10.1155/2010/178069

**Published:** 2010-02-01

**Authors:** Mones S. Abu-Asab, Majid Laassri, Hakima Amri

**Affiliations:** ^1^Laboratory of Pathology, National Cancer Institute, National Institutes of Health, Bethesda, MD 20892, USA; ^2^Laboratory of Methods Development, Center for Biologics Evaluation and Research, Food and Drug Administration, Rockville, MD 20852, USA; ^3^Department of Physiology and Biophysics, School of Medicine, Georgetown University, Washington, DC 20007, USA

## Abstract

Posttrial assessment of a vaccine's selective pressure on infecting strains may be realized through a bioinformatic tool such as parsimony phylogenetic analysis. Following a failed gonococcal pilus vaccine trial of *Neisseria gonorrhoeae*, we conducted a phylogenetic analysis of pilin DNA and predicted peptide sequences from clinical isolates to assess the extent of the vaccine's effect on the type of field strains that the volunteers contracted. Amplified pilin DNA sequences from infected vaccinees, placebo recipients, and vaccine specimens were phylogenetically analyzed. Cladograms show that the vaccine peptides have diverged substantially from their paternal isolate by clustering distantly from each other. Pilin genes of the field clinical isolates were heterogeneous, and their peptides produced clades comprised of vaccinated and placebo recipients' strains indicating that the pilus vaccine did not exert any significant selective pressure on gonorrhea field strains. Furthermore, sequences of the semivariable and hypervariable regions pointed out heterotachous rates of mutation and substitution.

## 1. Introduction

The recent failure of the HIV vaccine's STEP Study is a reminder that there is not usually an apparent reason that may explain a trial's demise [1, 2]. Only basic research will provide an understanding of why a vaccine had not worked and guidance for the design of better candidates [2]. As a step in this direction, we sought to provide a bioinformatic tool that is capable of gauging whether a vaccine has exerted any selective pressure on infectious field strains, as this may aid in reformulating the vaccine or the design of other candidates. A comparative algorithmic model for establishing the extent of a vaccines' efficacy is currently lacking although it may contribute to the improvement of formulation and implementation of future vaccine hypotheses. 

We are presenting a new analytical model that applies the principles of phylogenetics, such as parsimony, to assess whether a vaccine has affected the selection of infectious strains during a trial. Our approach relies on the robust parsimonious modeling of fast arising genetic variation to discriminate between two groups that are under different selective pressures [3, 4]. If a vaccine is shown to exert a selective pressure, then its formulation can be modified to broaden its effective range. Although phylogenetic algorithms have been applied in the classification of microorganisms and to detect recombination in a multiple sequence alignment, they have not been used in vaccine trial assessment [5, 6].

This study is a follow up on a field trial conducted among U.S. personnel stationed in the Republic of South Korea [7]. For the trial, a purified pilus preparation was isolated from Pgh 3-2* Neisseria gonorrhoeae* strain and tested as a vaccine in 3123 men and 127 women volunteers [7, 8]. Among male volunteers, 108 vaccine and 102 placebo recipients contracted gonorrhea after 15 or more days following vaccination. None of the women volunteers developed gonococcal infections. Samples of clinical isolates from all infected participants were plated on selected media, identified, and stored at the Department of Bacterial Diseases (Walter Reed Army Institute of Research, Washington, DC, USA). The authors of the trial concluded that the pilus vaccine failed to protect men against gonococcal urethritis during the field trial [7].

Gonococcal type IV pilus is filamentous proteinaceous surface structure responsible for initial bacterial attachment and is associated with virulence of *N. gonorrhoeae* (the gonococcus) [9, 10]. The pilus is a polymer comprised of pilin subunits; the latter share a common distinctive structure that also occurs in the pilins of other genera and is termed T4 pilin. The T4 pilin of *N. gonorrhoeae* is comprised of a highly conserved domain (C: 1–53 amino acids), a semivariable domain (SV: 54–114 amino acids), a hypervariable region (HV: variable number of amino acids) flanked by two conserved regions with each containing a cysteine residue, and a variable COOH-terminal region of irregular length following the second cysteine region [11].

Genetic variation that occurs at the SV and HV regions of the pilin involves a multigene system and has antigenic implications [12, 13]. Within a gonococcal genome, a structural gene (*pilE*) encodes for the pilin subunits. In addition to *pilE*, the genome contains several silent pilin genes (*pilS*); each *pilS* has one or more incomplete pilin gene(s) arranged in tandem and connected by intervening sequences [14]. Partial pilin copies of *pilS* lack the conserved region of *pilE* but have the same arrangement of SV and HV [14]. Recombination events between silent and expressed sites result in variations in the expressed pilin [15]. Thus, *pilE* replaces some, but not all, of its variable sites from any of the silent copies. 

The most suitable method for analyzing fast arising mutations, such as those in the SV and HV regions of the pilin, is sequencing followed by a parsimony phylogenetic analysis [3]. Our analysis examines the pilin composition of the vaccine and several clinical isolates from the vaccine trial to assess whether the vaccine had any selective effect on field strains that infected the vaccinated participants in spite of its failure to protect participant from infection. We applied a maximum parsimony phylogenetic algorithm to classify the pilin sequences according to their phyletic relatedness [3, 4], which has the capability to model a fast changing DNA and recent divergence of genes better than maximum likelihood or clustering [3].

## 2. Materials and Methods

### 2.1. Vaccine Strains and Clinical Isolates

Bacterial strains from the vaccine trial were obtained from the depository of the Department of Bacterial Diseases at Walter Reed Army Institute of Research, Washington, DC, where they were kept at −80°C [7]. To our knowledge, the vaccine strain did not undergo any further passages since vaccine preparation and this study. All the isolates used in this work were chosen randomly from positive samples; 40 isolates coded from 1 to 40 were used for hybridization analysis, and 12 strains (Table 1) were used for the sequencing of their pilin gene. Although the number of trial strains included in the sequencing and phylogenetic analysis was restricted to 12 strains, it was still sufficient to test our hypothesis. 

### 2.2. Pilin Gene Amplification

Bacteria cells from the frozen stock were used without subculture and lysed by heating in 100 *μ*L of 5% Chilex (Bio-Rad, Hercules, CA) for 5 min at 95°C. For PCR, 5 *μ*L of the Chilex solution was used. Primer selection was based on published sequences of pilin genes [16, 17]; forward (TACATTGCATGATGCCGATGG) and reverse (CGTTCCGCCCGCCCCAGCAGGC) primers amplified only the expressed pilin gene (*pilE*) and not the silent homologous copies.

### 2.3. Hybridization Experiments

To detect whether the expressed pilin genes from the isolated field strains were homologous or heterologous to that of the vaccine strain, P32brntn, the strains' amplicons were probed with oligonucleotides corresponding to the semivariable (SV) segments and the hypervariable (HV) regions of the vaccine pilin. Based on the pilin sequences of P32brntn, oligonucleotides corresponding to variable segments of the SV (GCTTTCAAAAATCAT and CAAATGGCTTCAAGCAA) and the total lengths of the HV (CCGACAACGACGACGTCAAA and GAGGCCGCCAACAACGGC) were synthesized and labeled with S^35^ isotope. Pilin gene amplicons from the 40 trial isolates were downloaded on a nylon membrane and probed with the synthetic oligonucleotides. The hybridizations were carried out at different stringency levels (50, 46, and 42°C) to detect the presence of closely homologous sequences and the degree of heterogeneity within the field strains.

### 2.4. Amplicons Cloning, Sequencing, and Translation

The PCR-produced amplicons of pilin gene from 12 strains (Table 1) were cloned into an M13 vector (Applied Biosystems, Foster City, CA). The cloned pilin genes were sequenced and translated into their predicted amino acids using GeneDoc [18].

### 2.5. Parsimony Phylogenetic Analysis

We used Protpars from the PHYLIP package to carry out the parsimony phylogenetic analysis [19]. Three sets of parsimony analyses, using amino acid sequences, were carried out: first, the whole sequence of the gene; second, SV regions alone (amino acids 51–127); and third, HV regions alone (amino acids 109–166). The latter two regions were analyzed to find out whether their sequences produced similar results to that of the whole sequence and whether the two regions' phylogenies were congruent with each other. This provided a test for the strain-specific pilin hypothesis since different regions of the peptides should not produce substantially varying hypotheses of relationships if the pilin is strain specific. 

## 3. Results

First, the variability of the *pilE* gene in 40 clinical isolates was analyzed by hybridization with the selected oligonucleotide probes of the vaccine strain, P32brntn. The results were negative at all stringency levels. This indicated the absence of homologous or partially homologous pilin genes in the clinical isolates of infected participants.

**Figure 1:**
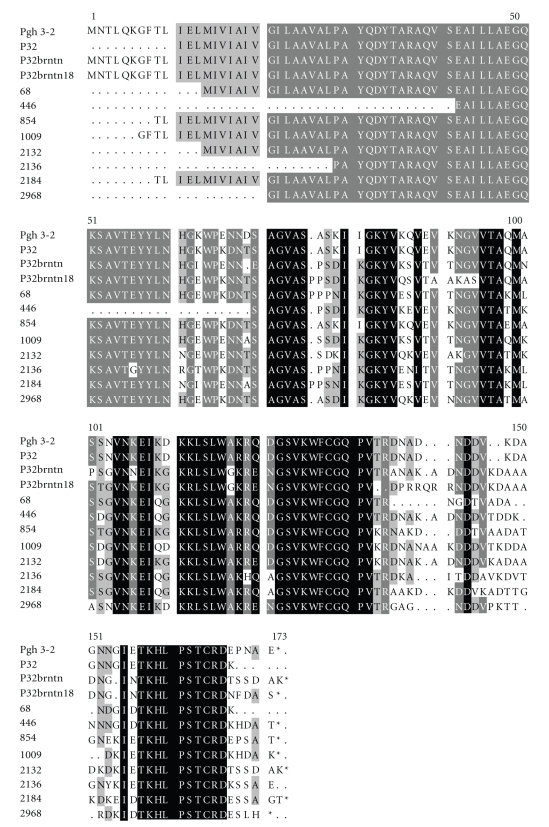
Multiple sequence alignment of pilus predicted peptides from 12 strains used in the analysis (Table 1). These peptide sequences were produced from translating DNA sequences (see Table 1 for GenBank accession numbers). There are three domains in the pilus peptide: conserved domain (C: 1–53 amino acids), a semivariable domain (SV: 54–114 amino acids), and a hypervariable region (HV: variable number of amino acids starting at amino acid 132). The color shadings (white, gray, and black) indicate the variability of the sequence; we have white: high variability, gray: slightly variable, and black: highly conserved.

**Figure 2:**
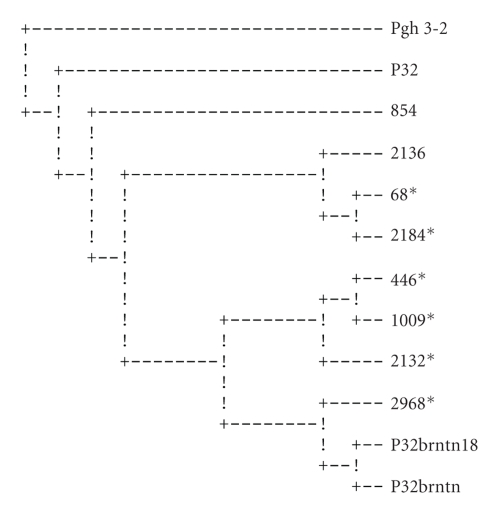
Most parsimonious cladogram of full-length predicted peptides. Pgh 3-2 was used as an outgroup since it is the ancestral strain of the vaccine strains. Strains from infected vaccinees are marked by ∗. For a few strains, small sequence segments at the beginning of the gene were not obtained and were treated as missing values in the analysis.

**Figure 3:**
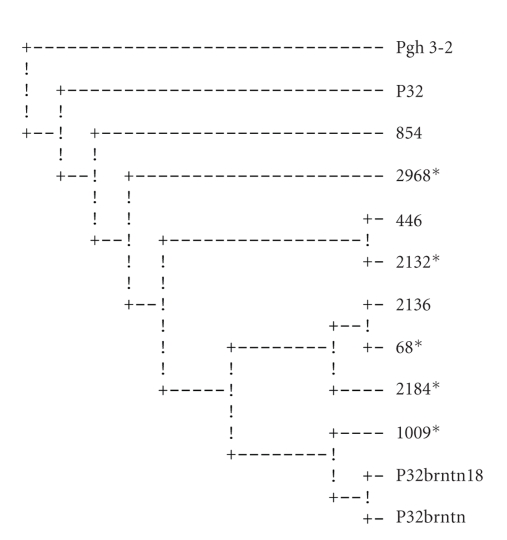
Consensus cladogram of the semivariable (SV) regions peptides (included amino acids 51–127). Pgh 3-2 was used as an outgroup. Strains from vaccinated individuals are marked by *.

**Figure 4:**
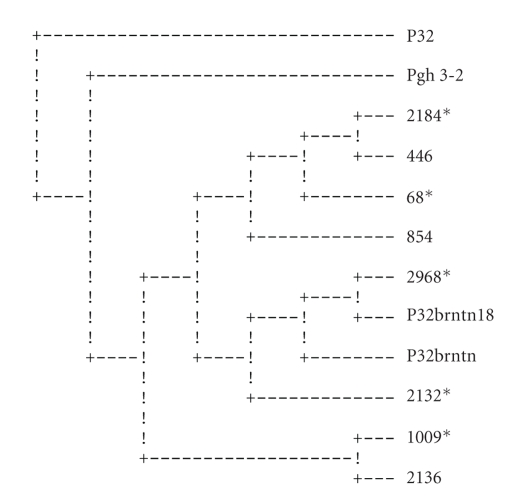
Consensus cladogram of the hypervariable (HV) regions peptides (included amino acids 109–166). Pgh 3-2 was used as an outgroup. Strains from vaccinated individuals are marked by *.

To confirm this result, the sequencing analysis was performed for the 12 samples presented in Table 1, including the vaccine strain. We found that the vaccine strain contained two pilin gene sequences (P32brntn and P32brntn18, Table 1, Figure 1) instead of one pilin gene sequence as it was thought by the authors of the trial [7]. These two pilins seem very closely related as they grouped together in three different cladograms (Figures 2–4). 

The sequences of the 12 specimens used in the study were congruent with the published structure of *Neisseria* pilins (GenBank accession numbers are listed in Table 1). However, the SV and HV regions (DNA and peptide sequences) of field strains were dissimilar to those of the vaccine (Figure 1). The variation among the sequences is shown phenetically (i.e., overall similarity, Figure 1), and phylogenetically (their phyletic relatedness, Figures 2–4).

Maximum parsimony analysis with Protpars [19] using whole peptide sequences produced one parsimonious cladogram (Figure 2). The SVs produced 4 equally parsimonious cladograms (Figure 3 shows the consensus cladogram); the HVs produced 12 equally parsimonious cladograms (Figure 4 shows the consensus cladogram).

All three parsimony phylogenetic analyses did not assemble separate groups for the strains isolated from vaccinees cohort and those isolated from placebo recipients. The strains of both groups were very closely related. This suggests that the vaccine had no immunological selective pressure on the isolates.

## 4. Discussion

Postvaccine trial analysis beyond success or failure is a rarity due to lack of analytical methods. We are not aware of any existing models for carrying out such an analysis. As the HIV vaccine STEP Study has shown, a vaccine failure sometimes is an enigma and no obvious reasons are at hand to explain its failure [1, 2]. However, we are attempting here to introduce parsimony phylogenetic analysis as an analytical paradigm for posttrial examination (it may also be used for the formulation of future vaccine candidates). There are several goals of such analysis: first, to assess the heterogeneity of field strains in relation to vaccine strains; second, to evaluate the phyletic relationships among all the strains; and third, to find out if the vaccine exerts any immunological selective pressure at the gene level of the field strains that may affect the type of infecting strain.

The pilin gene sequence was not known at the time of the vaccine trial, and attempts to sequence the pilus peptide's subunits were not completely successful. Our sequencing results from the stored P32brntn strain revealed two distinct *pilE* genes indicating that the culture has some heterogeneity (P32brntn and P32brntn18, Table 1, Figure 1), which is in contrast with the assumption of the vaccine trial authors of a single-type pilus [7]. The exact composition of the vaccine is significant (whether it was a single-type or multiple-type pilus) in order to assess its implications on the outcome of the trial. 

The efficacy of a pilus vaccine in preventing gonorrhea infections was the subject of a long debate fueled by contradicting evidence [7, 20]. On one hand, the pili are associated with gonorrhea's virulence [21]; pilus vaccines have been effective in protecting suckling piglets and cattle against infections of *E. coli* and *Moraxella bovis*, respectively [7, 22]; and these vaccines were immunogenic [23]. On the other hand, the pilus vaccine was ineffective beyond the homology of its pilus strain and even its homologous protection was overcome with larger challenge inocula [20]. The authors of the vaccine trial argued that human challenge experiments do not always predict the outcome in a natural setting and embarked on a large placebo-controlled, double-blinded field trial of pilus vaccine [7]. Although the vaccine elicited a good immune response in vaccinated recipients, it failed to protect them [24]. This work examined the extent of pilin diversity among infected participants and pilin phylogeny as indicators of the vaccine selective pressure. We explored a new analytical model to determine whether vaccine effectiveness can be assessed on the basis of pilin sequences phylogeny to infer whether the vaccine exerted a selective pressure on the gonorrhea strains that infected the vaccinated participants.

The heterogeneity of the vaccine inoculum (two pilin types: P32brntn and P32brntn18) did not seem to confer any additional effectiveness on the vaccine. This could be attributed to the close sequence similarity of the two; the two types have shared sequences and grouped together in all three sets of the analyses (Figures 2–4). 

In order to test the validity of our hypothesis, which is based on the phylogeny of the pilin genes, the ancestral strains, Pgh 3-2, a clinical isolate from which the vaccine strain was derived [8], and a strain derived from it (P32), were sequenced and included in the analyses. The two ancestral strains clustered together in all three cladograms (Figures 2–4), while the vaccine pilins clustered distantly from them. On two of the cladograms, the ancestral pilin and the vaccine pilins were separated from each other by all the other isolates (Figures 2 and 3). The vaccine pilins consistently paired with the participants' sequences. Since the phylogenetic history of these strains is well known to us, one can conclude that the pilin sequences of the vaccine have diverged from their ancestral strains to a point where their true phylogeny is not reflected in their pilin sequences. Furthermore, it seems that because of recombination events as well as high mutation rate, particularly at the HV region, a strain-specific pilin appears to be an inaccurate term.

The phylogenetic analysis seems to indicate that the vaccine did not appear to have influenced the strain type in the vaccinated group. This is inferred from the groupings of the sequences of the placebo and vaccinated groups where they appear together in mixed groups (Figures 2–4). If the vaccine had any selective pressure against gonorrhea strains, the placebo and vaccinees groups would have been expected to group separately from one another on the cladograms.

This study provided a clear insight into the magnitude of antigenic variation of pilin exhibited among field strains, and therefore, permits an evaluation of the feasibility of pili as a vaccine against one of the highest reported infections in the US—gonorrhea [25]. This high heterogeneity of pilin provides a strong reasoning against a single-type pilus vaccine and lends support for multitype pilus of future vaccine candidates.

Variation within the expressed pilin gene is partially derived from intragenomic recombination events between the former and copies of silent pilin genes *pilS* [26]. Therefore, in light of the results obtained from phylogenetically assessing the three segments of pilin gene (Figures 2–4), it will be important to assess the degree to which silent copies in the clinical isolates have contributed to the variation within expressed pilin gene. This step is postponed for a future study.

## Figures and Tables

**Table 1:** Strains of *Neisseria gonorrhoeae* used in the study.

Strains	Origin	GenBank Accessions
Pgh 3-2	clinical isolate [8]	EU379154
P32	derived from Pgh 3-2 [7]	EU379152
P32brntn	vaccine strain derived from Pgh 3-2	EU379153
P32brntn18	vaccine strain derived from Pgh 3-2	U16742
68	from a vaccinated participant	EU340030
1009	from a vaccinated participant	EU360770
2132	from a vaccinated participant	EU379148
2184	from a vaccinated participant	EU379150
2968	from a vaccinated participant	EU379151
446	from a placebo recipient	EU346893
854	from a placebo recipient	EU360769
2136	from a placebo recipient	EU379149
